# Behavioral Impairments and Oxidative Stress in the Brain, Muscle, and Gill Caused by Chronic Exposure of C_70_ Nanoparticles on Adult Zebrafish

**DOI:** 10.3390/ijms20225795

**Published:** 2019-11-18

**Authors:** Sreeja Sarasamma, Gilbert Audira, Prabu Samikannu, Stevhen Juniardi, Petrus Siregar, Erwei Hao, Jung-Ren Chen, Chung-Der Hsiao

**Affiliations:** 1 Department of Chemistry, Chung Yuan Christian University, Chung-Li 32023, Taiwan; sreejakarthik@hotmail.com (S.S.); gilbertaudira@yahoo.com (G.A.); chemistprabu5590@gmail.com (P.S.); 2 Department of Bioscience Technology, Chung Yuan Christian University, Chung-Li 32023, Taiwan; stvn.jun@gmail.com (S.J.); siregar.petrus27@gmail.com (P.S.); 3 Guangxi Key Laboratory of Efficacy Study on Chinese Materia Medica, Guangxi University of Chinese Medicine, Nanning 530200, China; 4 Guangxi Collaborative Innovation Center for Research on Functional Ingredients of Agricultural Residues, Guangxi University of Chinese Medicine, Nanning 530200, China; 5 Department of Biological Science & Technology College of Medicine, I-Shou University, Kaohsiung 82445, Taiwan; 6 Center for Biomedical Technology, Chung Yuan Christian University, Chung-Li 32023, Taiwan; 7 Center for Nanotechnology, Chung Yuan Christian University, Chung-Li 32023, Taiwan

**Keywords:** C_70_, reactive oxygen species, nanoparticle toxicity, behavior tests, phenomics

## Abstract

There is an imperative need to develop efficient whole-animal-based testing assays to determine the potential toxicity of engineered nanomaterials. While previous studies have demonstrated toxicity in lung and skin cells after C_70_ nanoparticles (NPs) exposure, the potential detrimental role of C_70_ NPs in neurobehavior is largely unaddressed. Here, we evaluated the chronic effects of C_70_ NPs exposure on behavior and alterations in biochemical responses in adult zebrafish. Two different exposure doses were used for this experiment: low dose (0.5 ppm) and high dose (1.5 ppm). Behavioral tests were performed after two weeks of exposure of C_70_ NPs. We found decreased locomotion, exploration, mirror biting, social interaction, and shoaling activities, as well as anxiety elevation and circadian rhythm locomotor activity impairment after ~2 weeks in the C_70_ NP-exposed fish. The results of biochemical assays reveal that following exposure of zebrafish to 1.5 ppm of C_70_ NPs, the activity of superoxide dismutase (SOD) in the brain and muscle tissues increased significantly. In addition, the concentration of reactive oxygen species (ROS) also increased from 2.95 ± 0.12 U/ug to 8.46 ± 0.25 U/ug and from 0.90 ± 0.03 U/ug to 3.53 ± 0.64 U/ug in the muscle and brain tissues, respectively. Furthermore, an increased level of cortisol was also observed in muscle and brain tissues, ranging from 17.95 ± 0.90 pg/ug to 23.95 ± 0.66 pg/ug and from 3.47 ± 0.13 pg/ug to 4.91 ± 0.51 pg/ug, respectively. Increment of Hif1-α level was also observed in both tissues. The elevation was ranging from 11.65 ± 0.54 pg/ug to 18.45 ± 1.00 pg/ug in the muscle tissue and from 4.26 ± 0.11 pg/ug to 6.86 ± 0.37 pg/ug in the brain tissue. Moreover, the content of DNA damage and inflammatory markers such as ssDNA, TNF-α, and IL-1β were also increased substantially in the brain tissues. Significant changes in several biomarker levels, including catalase and malondialdehyde (MDA), were also observed in the gill tissues. Finally, we used a neurophenomic approach with a particular focus on environmental influences, which can also be easily adapted for other aquatic fish species, to assess the toxicity of metal and carbon-based nanoparticles. In summary, this is the first study to illustrate the adult zebrafish toxicity and the alterations in several neurobehavior parameters after zebrafish exposure to environmentally relevant amounts of C_70_ NPs.

## 1. Introduction

As the industrial applications of stabilized nanoparticles continue to expand, it becomes crucial to understand their potential environmental implications. The potential application of nanomaterials includes drug delivery, medical equipment, biosensors, and personal care products [1]. The projected widespread use and large-scale production volume have led to growing concern over the potential for most engineered nanomaterials to adversely affect human health and the environment [2,3].

Nowadays, among carbon-based nanomaterials, fullerenes have received much attention with many potential applications, including in electronics, site-specific drug delivery, and pharmaceutical nanocarriers [4,5,6]. In fact, in the pharmaceutical industry, the potential antioxidant nature of fullerenes could be identified as a therapeutic intervention for the nervous system and neurodegenerative diseases, diabetes, pancreatic disease, skin damage, hearing loss, septic shock, and kidney diseases. Fullerenes are being marketed to consumers as a therapeutic agent in cosmetics and creams [7]. In addition, fullerenes can serve as major components in a variety of plastics, including filtration membranes [8]. However, many studies are raising safety concerns by demonstrating possible cytotoxic effects of fullerenes and their derivatives [9,10,11,12,13]. With increasing large production volume and use of fullerene, it is imperative to determine/monitor the possible human health and environmental implications [14] of these nanomaterials since adverse effects as well as protective effects are reported upon exposure [15,16,17]. Lack of toxicological data on nanomaterials makes it difficult to determine if there is a risk connected with nanoparticle exposure. Timely assessment of nanoparticle toxicity would provide this critical data, enhance the public trust of the nanotechnology industry, and aid regulators in deciding the environmental and health risks of commercial nanomaterials [18]. Hence, there is an urgent need to develop efficient, rapid, and appropriate testing strategies to assess toxicity and potential risks posed by fullerenes and their derivatives.

Various biological models have been used for the toxicological assessment of nanomaterials. In vitro techniques, such as cell culture, are often used because they are efficient, rapid, and less expensive [15]. While these in vitro studies are useful, a direct translation to human health risk is often difficult to understand [19]. In vivo studies, on the other hand, can provide improved prediction of the biological response in an intact system. Since in vivo studies often employ rodent models, assessments are generally time-consuming, expensive, and require extensive facilities for housing experimental animals. Time, labor, infrastructure, and cost can be significantly reduced by replacing the traditional rodent model with the zebrafish model [20]. Zebrafish are a well-established model for studying toxicological assessments and basic developmental biological processes [21,22,23,24]. Currently, only a handful of studies have investigated the adverse effects of C_70_ NPs on *Daphnia* [25] but there is a paucity of studies on adult zebrafish. Furthermore, the cellular and biochemical mechanisms underlying C_70_ NP toxicity are still poorly understood.

A number of diverse platforms are available to assess toxicity, ranging from in vitro studies to basic model organisms, such as *Daphnia* or sea urchins, to higher vertebrate models, such as rodents and primates [26,27]. Recent studies have begun to apply “big data” approaches to aid in data analysis and interpretation for validation of drugs and behaviors in zebrafish [28,29]. In this context, zebrafish behavioral phenomics are emerging as a new platform directed towards assessing various behavioral phenotypes by means of high-throughput screening and test batteries [30]. This new area of zebrafish phenomics-based biology is gaining importance in aquatic toxicology and neuropharmacology, in addition to the search for genes and pathways that can serve as biomarkers or targets for drug exposure.

**Figure 1:**
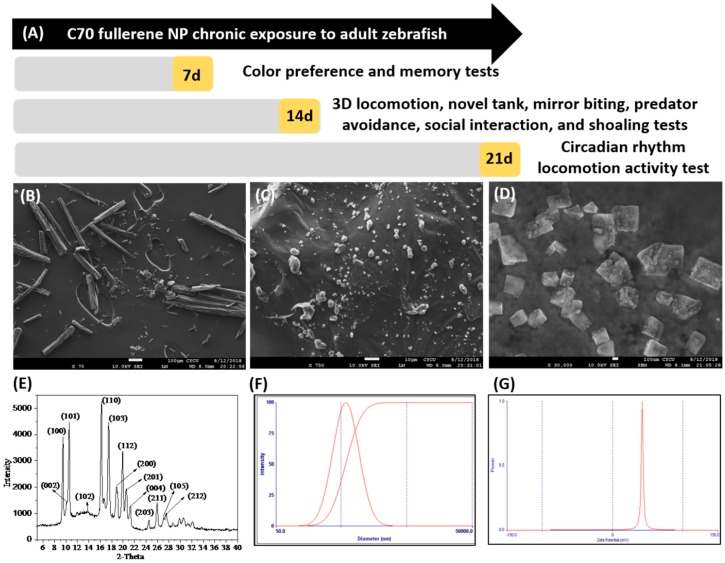
(**A**) Overview of the experimental design and time points for chronic exposure of C_70_ fullerene nanoparticles (NPs) to adult zebrafish. For chronic toxicity, we measured color preference and short-term memory at 7 days post-exposure (dpe). 3D locomotion, novel tank, mirror biting, predator avoidance, social interaction, and shoaling tests were given at 14 dpe. The circadian rhythm test was given at 21 dpe. After all behavior tests, fish were dissected and subjected to biochemical assays by 22 dpe. Characterization of the C_70_ NPs used in this study: (**B**) SEM micrograph of C_70_ NPs stock solution in the absence of solvents, (**C**) C_70_ NPs dissolved in DMSO showing wide disparity in aggregation, (**D**) high magnification scanning electron micrograph showing the size of C_70_ NPs used in this study, and (**E**) X-ray diffraction patterns of the crystal quality of the C_70_ NPs. (**F**) The particle size distribution of 0.5 ppm C_70_ NPs in DMSO was measured by dynamic light scattering. C_70_ NP suspensions were sonicated prior to measurement to resuspend the large particles and assess changes in large aggregate status. (**G**) The zeta potential value of C_70_ NPs is estimated at −34.0 mV.

In this continuum, a small number of reports deal with possible toxicities of C_70_ NPs with the aid of either in vitro or in vivo studies [31,32], but their neurobehavior impairments were not definitively determined. No evidence was currently available to allow for predictions of behavioral functions that would most likely be affected by these nanomaterials. There is an urgent need for a molecular biomarker that would be used as an endpoint to evaluate neurobehavior toxicities. To this end, this study aimed to investigate the effects of toxicity level and stress response of adult zebrafish to fullerene C_70_ NPs. To understand the mechanism underlying the abnormal neurobehavior and the oxidative inflammation in the brain caused by nanoparticulate C_70_, we investigate the different endpoints of behavior analysis and the pathological changes in various tissues following exposure to zebrafish, assess the oxidative stress markers, and examine the effects of neurotransmitters including γ-aminobutyric acid (GABA), acetylcholinesterase (AChE) activity, and levels of dopamine (DA), serotonin (5-HT), and melatonin in the zebrafish brain. The experimental design and times for behavioral endpoint measurement were summarized in Figure 1A.

## 2. Results

### 2.1. Physical Property Characterization of C_70_ NPs (Nanoparticles)

The size of C_70_ NPs was determined by X-ray diffraction (XRD) analysis and scanning electron microscopy (SEM). As shown in Figure 1B, the nanoparticles appear mainly spherical. Due to the insolubility of C_70_ NPs in water, it was sonicated to form a uniform suspension in 0.1% of dimethyl sulfoxide (DMSO). Previous evaluations in our laboratory have demonstrated no adverse biological effect of DMSO at this concentration [33]. The SEM data showed crystalline particles of C_70_ NPs with a diameter of 95.02 ± 0.25 nm (Figure 1D). The crystal structure of C_70_ NPs was analyzed by XRD and showed intense diffraction peaks. In Figure 1E, the XRD patterns of C_70_ NPs peaks at 2θ = 9.5°, 11.9°, 14.3°, 16.2°, 18.5°, 19.6°, 21.5° were assigned to (100), (101), (102), (110), (103) respectively (Figure 1E). The following suspension at DMSO C_70_ nanoparticles size distribution was measured by dynamic light scattering (DLS) (Figure 1F). Zeta potential measurements, evaluated by electrophoretic mobility of C_70_ nanoparticle in Figure 1G, indicated that it acquired a negative surface charge of −34.0 mV.

### 2.2. Low-Dose Exposure of C_70_ NPs Reduced Locomotion and Exploration Behaviors

**Figure 2:**
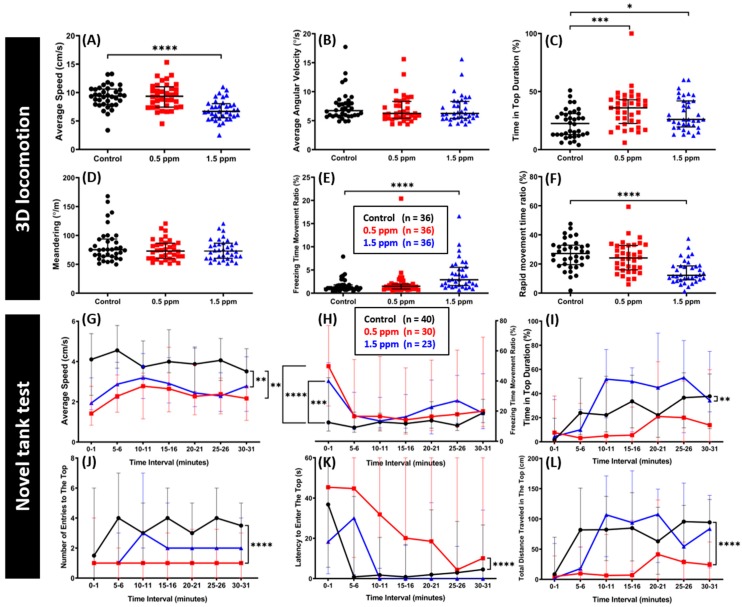
Comparison of behavior endpoints between the untreated control and C_70_ NP-exposed zebrafish in 3D locomotion and novel tank tests after 14-day exposure. (**A**) Average speed, (**B**) average angular velocity, (**C**) time in top duration, (**D**) meandering, (**E**) freezing time movement ratio, and (**F**) rapid movement time ratio were analyzed for the 3D locomotion test. For the novel tank test, (**G**) average speed, (**H**) freezing time movement ratio, (**I**) time in top duration, (**J**) number of entries to the top, (**K**) latency to enter the top, and (**L**) total distance traveled in the top were analyzed. The data are expressed as the median with interquartile range. The 3D locomotion test data were analyzed by the Kruskal–Wallis test, with Dunn’s multiple comparisons test as a follow-up test (*n* = 36 for both control and treatment groups). The novel tank test data were analyzed by two-way ANOVA with Geisser–Greenhouse correction (*n* = 40 for the untreated control; *n* = 30 for the 0.5 ppm C_70_ NP-exposed fish; *n* = 23 for the 1.5 ppm C_70_ NPs-exposed fish; * *p* < 0.05, ** *p* < 0.01, *** *p* < 0.001, **** *p* < 0.0001).

The locomotor activity was assessed using a three-dimensional (3D) locomotion test assay in zebrafish after chronic exposure to two different concentrations of C_70_ NPs (0.5 and 1.5 ppm) for two weeks. In this test, we measured six important zebrafish behavior endpoints representative of swimming activity and orientation. From the results, we found that 1.5 ppm of C_70_ NP-treated fish showed a significant reduction in their locomotor activity. On another hand, a lower concentration of C_70_ NP_S_ (0.5 ppm) did not show any behavioral alteration in swimming activity. These phenomena were indicated by a lower average speed, a rapid movement ratio, and a higher freezing time ratio exhibited by zebrafish treated with 1.5 ppm C_70_ NP_S._ Furthermore, there were no significant differences in the average speed, freezing, and rapid movement time ratio between 0.5 ppm of C_70_ NP-treated fish and the control group (Figure 2A,E–F). In addition, similar average angular velocity and meandering were recorded in both treated fish and the control groups suggesting swimming orientation was not affected (Figure 2B,D). Interestingly, it was observed that both 0.5 and 1.5 ppm C_70_ NP-treated groups showed a significant increase in time of top duration compared to the control group (Figure 2C).

The novel tank test, another experiment to assess zebrafish locomotor activity, exploratory behavior, and anxiety level was conducted after two weeks of C_70_ NPs exposure. This test exploits the natural tendency of zebrafish to initially dive to the bottom part of a novel tank, with a gradual increase in activity in the vertical axis over time [34]. This test revealed adult fish exposed to both concentrations of C_70_ NPs showed a significant decrease in their swimming activity compared to the controls. This finding was shown by a lower average speed and a higher freezing time movement ratio of treated fish compared with the control group in most of the experiment time (Figure 2G,H). Furthermore, 0.5 ppm of C_70_ NP_s_ altered the exploratory behavior, indicated by a lower average of time in top duration, a lower level of entries to the top, and a lower total distance traveled in the top, and a higher level of latency to enter the top portion of the test tank (Figure 2I–L). Surprisingly, higher concentration of C_70_ NP_s_ did not cause a more severe effect in the exploratory behavior, as indicated by the similar average of time in top duration, number of entries to the top, total distance traveled in the top, and latency to enter the top portion of the test tank in most of the test time interval seen in the treated fish and the control group (Figure 2I–L).

### 2.3. Low Doses of C_70_ NPs Exposure Reduced Aggression and Predator Avoidance

**Figure 3:**
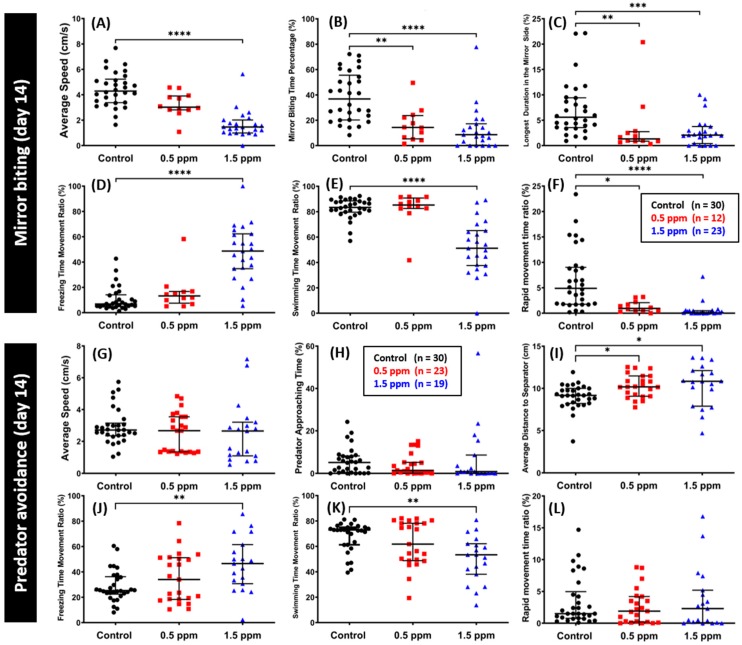
Comparison of mirror biting and predator avoidance behavior endpoints between the untreated control and C_70_-exposed fish after 14-day exposure. (**A**) Average speed, (**B**) mirror biting time percentage, (**C**) longest duration in the mirror side, (**D**) freezing time movement ratio, (**E**) swimming time movement ratio, and (**F**) rapid movement time ratio were analyzed for the mirror biting assay (*n* = 30 for the untreated control; *n* = 12 for the 0.5 ppm C_70_ NP-exposed fish; *n* = 23 for the 1.5 ppm C_70_ NP-exposed fish). For predator avoidance test, the (**G**) average speed, (**H**) predator approaching time ratio, (**I**) average distance to separator, (**J**) freezing time movement ratio, (**K**) swimming time movement ratio, and (**L**) rapid movement time ratio were analyzed (*n* = 30 for the untreated control; *n* = 23 for the 0.5 ppm C_70_ NP-exposed fish; *n* = 19 for the 1.5 ppm C_70_ NP-exposed fish). The data are expressed as the median with interquartile range and were analyzed by the Kruskal–Wallis test continued with Dunn’s multiple comparisons test as a follow-up test (* *p* < 0.05, ** *p* < 0.01, *** *p* < 0.001, **** *p* < 0.0001).

To measure the aggressiveness of the fish, the mirror biting test was conducted after two weeks of C_70_ NPs exposure. Biting the mirror may also indicate, more generally, social motivation or the intent to interact with a social partner [15]. In this test, chronic exposure of C_70_ NPs in both concentrations significantly reduced zebrafish aggressiveness, as indicated by a lower mirror biting time percentage and the longest duration in the mirror side (Figure 3B–C). Furthermore, in line with our previous C_60_ NP_S_ studies [15], 1.5 ppm of C_70_ NPs exposure reduced their locomotion behavior, displayed by a lower average speed, swimming and rapid movement time ratios, and a higher freezing time movement ratio compared to the control fish (Figure 3A,D–F). In addition, a slight decrement in locomotor activity was also detected in 0.5 ppm of C_70_ NP-treated fish, as shown by a lower rapid movement time ratio (Figure 3F). Meanwhile, there was no significant difference found in other types of fish movement and average speed between 0.5 ppm of C_70_ NPs treated and the control group (Figure 3A,D–E).

Fear is a collection of behavioral responses that are elicited by negative stimuli associated with imminent danger such as the presence of a predator [35]. In the predator avoidance test, we assessed zebrafish fear reactions, including anti-predatory behavior after two weeks of C_70_ NPs exposure. Predator avoidance analysis was conducted by confronting zebrafish with a predator, convict cichlid (*Amatitlania nigrofasciata*). We found that exposure of C_70_ NPs in zebrafish did not alter their fear response behavior to their predator, showed by similar predator approaching time between the treated zebrafish and the control (Figure 3H). Meanwhile, there were slight increments in the average distance to separator between zebrafish and the predator fish exhibited by both the 0.5 ppm and the 1.5 ppm C_70_ NP-treated zebrafish (Figure 3I). Furthermore, we found some differences in the types of movement in the 1.5 ppm of C_70_ NP-treated zebrafish compared with the control fish, even though their average speed was similar (Figure 3G). These differences were indicated by a higher freezing time movement ratio and a lower swimming time movement ratio, while there was no significant difference in their rapid movement time ratios (Figure 3J–L).

### 2.4. Low Doses of C_70_ NPs Exposure Dysregulated Social Interaction and Shoaling Behavior

**Figure 4:**
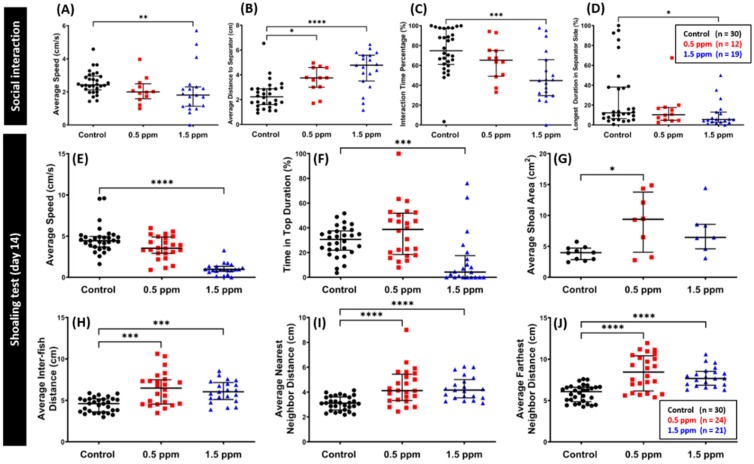
Comparison of social interaction and shoaling behavior endpoints between the untreated control and C_70_ NP-exposed fish after 14-day exposure. (**A**) Average speed, (**B**) average distance to separator side, (**C**) interaction time percentage, and (**D**) the longest duration in the separator side were analyzed for the social interaction test (*n* = 30 for the untreated control; *n* = 12 for the 0.5 ppm C_70_ NP-exposed fish; *n* = 19 for the 1.5 ppm C_70_ NP-exposed fish). For the shoaling test: (**E**) average speed, (**F**) time in top duration, (**G**) average shoal area, (**H**) average inter-fish distance, (**I**) average nearest neighbor distance, and (**J**) average farthest neighbor distance were analyzed (*n* = 30 for the untreated control; *n* = 24 for the 0.5 ppm C_70_ NPs-exposed fish; *n* = 21 for the 1.5 ppm C_70_ NPs-exposed fish, with 3 fish for each shoal). The data are expressed as the median with interquartile range and were analyzed by the Kruskal–Wallis test, with Dunn’s multiple comparisons test as a follow-up test (* *p* < 0.05, ** *p* < 0.01, *** *p* < 0.001, **** *p* < 0.0001).

The social interaction test is another useful model to study fish social phenotypes. It encompasses more than simply preferring to be near members of the same species as humans [15]. We assessed zebrafish sociability by observing zebrafish interactions with conspecific after two weeks of C_70_ NPs exposure [36]. Based on the results, we found that zebrafish chronically exposed to 1.5 ppm of C_70_ NPs had reduced conspecific social interaction, as shown by a lower interaction time percentage and a higher average distance to the separator and the longest duration in the separator side (Figure 4B–D). On another hand, low concentration of C_70_ NPs did not alter zebrafish conspecific interaction behavior, as shown by similar interaction time percentage and the longest duration in the separator side between the C_70_ NPs treated fish and untreated control, even though there was an increment in the average distance to the separator was noted in the 0.5 ppm C_70_ NPs treated fish (Figure 4B–D). Furthermore, locomotor activity alteration was detected in the high concentration of C_70_ NP-treated fish, which was shown by the low level of average speed while there was no similar phenomenon displayed by the low concentration of C_70_ NPs group, as shown by similar average speed compared to the control group (Figure 4A).

Shoaling behavior represents the complex interaction of animals moving together in coordinated movements which is very common in fish models [37]. We tested the social behavior of C_70_ NP-exposed fish using a shoaling assay after two weeks of C_70_ NPs exposure. In this test, loose shoals formed by C_70_ NP-treated fish were observed, as indicated by increased average shoal area, average inter-fish distance, average nearest neighbor distance, and average farthest neighbor distance in both C_70_ NPs treated groups (Figure 4G–J). Furthermore, in line with our previous C_60_ NPs studies [38], a significant decrease in locomotor activity was observed in the high concentration of C_70_ NPs compared to untreated control (Figure 4E). In addition, a high concentration of C_70_ NPs exposure also reduced the exploratory behavior as shown by decreased time in the top duration (Figure 4F).

### 2.5. Low Doses of C_70_ NPs Exposure Dysregulated the Color Preference

**Figure 5:**
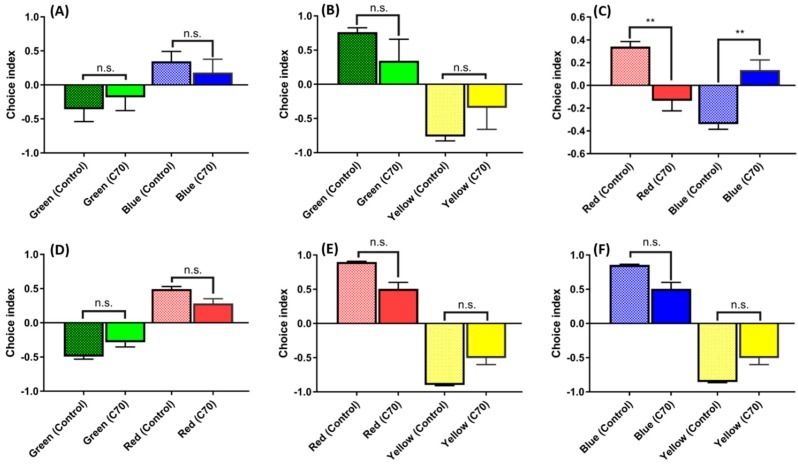
Comparison of color preferences between the untreated control and 1.5 ppm of C_70_ NP-exposed fish: (**A**) green vs. blue combination; (**B**) green vs. yellow combination; (**C**) red vs. blue combination; (**D**) green vs. red combination; (**E**) red vs. yellow combination; and (**F**) blue vs. yellow combination. Since all of the data were not normally distributed, they were analyzed using non-parametric Kruskal–Wallis followed by Dunn’s post-hoc test, and *p* < 0.05 was considered significantly different. The data are presented with mean ± SEM with *n* = 24, n.s. = not significant, ** *p* < 0.01.

Color vision is one of the most prominent modalities to recognize biologically-important stimulation and it plays a significant role in visual perception. Zebrafish possess eyes and retinas that are very similar to those of other vertebrates includes humans [38]. The color preference test has been used to assess phenotypical and behavioral changes in zebrafish [15,39,40]. Our previous color preference study showed that adult zebrafish exposed to C_60_ NPs have a strong aversion towards green/blue and red/blue compared to other colors [41]. The color preference patterns in C_70_ NP-treated fish showed changes in blue–red color combination after one-week exposure (Figure 5C), with overall preference pattern shifted from a red > blue > green > yellow preference in the untreated control to a blue > red > green > yellow preference in the C_70_ NP-treated fish. Other color combinations for either blue–green (Figure 5A), green–yellow (Figure 5B), red–green (Figure 5D), red–yellow (Figure 5E), or blue–yellow (Figure 5F) showed non-significant differences between the untreated control and the C_70_ NP-treated fish.

### 2.6. Low Doses of C_70_ NPs Exposure Dysregulated the Circadian Rhythm but Did Not Alter Short-Term Memory

**Figure 6:**
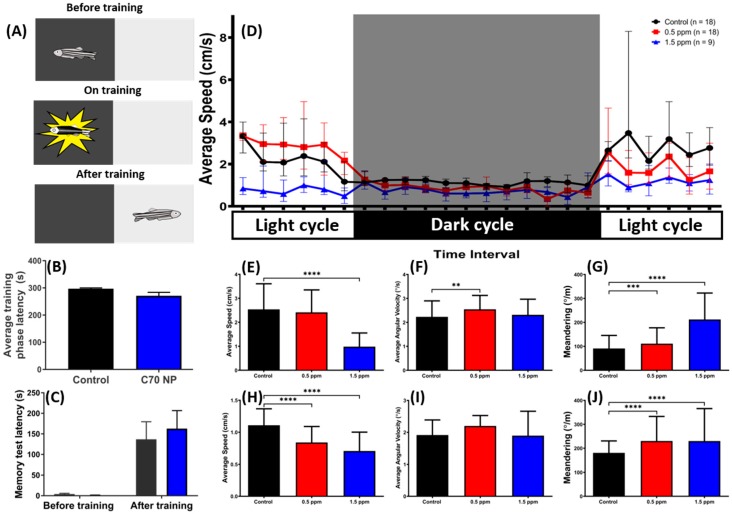
The short-term memory and circadian rhythm assay for the untreated control and C_70_ NP-exposed fish after 7- and 21-day exposure, respectively. (**A**) Schematic showing the experimental protocol for the passive avoidance test. (**B**) The average training phase latency on zebrafish learning. (**C**) The memory retention latency for the memory test. The data are expressed as the mean ± SEM and were analyzed by two-way ANOVA with Sidak’s multiple comparisons test as a follow-up test (*n* = 10 for the untreated control; *n* = 6 for the C_70_ NP-exposed fish). (**D**) Comparison of time chronological changes of the average speed between wild-type and C_70_ NP-exposed fish in the day and night cycle. The grey area shows the dark period and the unshaded area is the light period. Comparison of (**E**) average speed, (**F**) average angular velocity, and (**G**) meandering at light period. Comparison of (**H**) average speed, (**I**) average angular velocity and (**J**) meandering at the dark period. The data are expressed as the median with interquartile range and were analyzed by Kruskal–Wallis test, with Dunn’s multiple comparisons test as a follow-up test (*n* = 18 for the untreated control; *n* = 18 for 0.5 ppm C_70_ NP-exposed fish; *n* = 9 for the 1.5 ppm C_70_ NP-exposed fish; *** *p* < 0.001, **** *p* < 0.0001).

The zebrafish represents a potential model to study memory function and impairment in vertebrates. Here, we used a passive avoidance test to explore the potential short-term memory deficiency in zebrafish after three weeks exposed to environmental-level C_70_ NPs. This passive avoidance test was conducted by using a white–black shuttle box equipped with an electric shock following our previously published protocol (Figure 6A) [42]. However, no significant alteration on a short-term memory test in terms of either training-phase latency (Figure 6B) or testing-phase latency (Figure 6C) was found in the C_70_ NP-treated fish. This result demonstrated that chronic exposure to C_70_ NPs does not cause short-term memory alteration in zebrafish.

Light cycles are the most important synchronizers of biological rhythms in nature [15]. We assessed zebrafish circadian rhythms and the effects of light to dark photoperiods on zebrafish locomotor activity after three weeks of C_70_ NPs exposure (Figure 6D), represented as average swimming speed over time. In agreement with our other locomotor activity test results done in C_60_ NPs [42], high concentration of C_70_ NPs exposure-treated fish showed lower circadian locomotor activities than the control group during the light and dark periods, while low concentration of C_70_ NPs exposure only affected their locomotor activity during the dark cycle as indicated by abnormal level of average speed during both periods (Figure 6E,H). Furthermore, zig-zag-like movement behavior was also detected in C_70_ NP-treated groups, indicated by a higher level of meandering compared to the control group in both of the light and dark periods (Figure 6G,J). Meanwhile, there was no irregular swimming orientation exhibited by both treated fish groups, except for a high average angular velocity in the 0.5 ppm C_70_ NP-treated group during the light period (Figure 6F,I).

### 2.7. Impact of C_70_ NPs Exposure on Biomarker Expression in the Muscle, Brain, and Gill

By the end of all behavioral tests on day 22, we sacrificed the fish, dissected the muscle, brain, and gill tissues, extracted total proteins and subjected to perform an enzyme-linked immunosorbent assay (ELISA) to measure biomarker expression after C_70_ NP_S_ exposure. In muscle tissue, the effects of C_70_ NPs exposure on several important biomarkers such as reactive oxygen species (ROS) generation, lipid peroxidation (MDA and TBARS) production, and anti-oxidative stress enzyme (CAT and SOD) activities are shown in Table 1. The significant increases of ROS, TBARS and MDA contents were observed in the muscle after treatment of C_70_ NPs. While the increment of the MDA level was also shown in the gill tissue, there was no significant change in the treated fish gill regarding the TBARS level. The C_70_ NPs exposure also triggered the activation of antioxidative enzymes since we found the relative activities of CAT and SOD were significantly elevated in the muscle after C_70_ NPs exposure. This similar phenomenon was also shown in the gill tissues after the CAT activity was measured. By measuring the stress hormone, we provided evidence to show the strong anxiety behavior induced by C_70_ NP_S_ exposure was well agreed with the high elevation of cortisol level in the muscle. In addition, the hypoactivity in locomotion after C_70_ NP_S_ exposure led us to ask whether it was correlated to the muscle energy or oxygen supplement deficiency. We addressed this question by measuring the creatine kinase (CK), ATP and Hif1-α levels. Results showed the relative activity or content of CK and ATP was reduced in the muscle after C_70_ NP_S_ expose. The hypoxia marker of Hif1-α, on the contrary, displayed significantly elevation after C_70_ NP_S_ expose.

From the results, we also found that the activities of antioxidative enzymes in the brains from the 1.5 ppm and 0.5 ppm group were significantly lower than the control. Furthermore, the reduction of enzymatic activities in the brain caused by 1.5 ppm C_70_ NPs was greater than that by the 0.5 ppm and untreated controls. Combined with those findings, we concluded the oxidative stress in the muscle induced by the C_70_ NPs treatment was apparently more severe than the control group. The effects of C_70_ NPs exposure on DNA damage, hypoxia and inflammation were evaluated by measuring biomarkers such as ssDNA, Hif-1α, IL-1β, and TNF-α activity. In 0.5 ppm C_70_ NP-treated groups, the activities of inflammatory markers were not significantly different from those of the control. However, the activities of inflammatory markers in the brains and gills of 1.5 ppm C_70_ NP-treated groups were significantly higher than those of the control. Taken together, 1.5 ppm C_70_ NP-exposed zebrafish muscle and gill tissues showed a higher inflammatory marker response compared to the 0.5 ppm C_70_ NP-exposed groups and those of the untreated controls.

Considering that behavioral changes induced by C_70_ NPs exposure may be related to alterations on the cholinergic system, the effect of C_70_ NPs exposure on AChE, ACh, serotonin, and melatonin activity from the brain of zebrafish were evaluated. Data in Table 1 showed a significant inhibition of ACh, melatonin, and serotonin activities in the brain of the 1.5 ppm C_70_ NP-treated group. Moreover, AChE activity was significantly increased by C_70_ NPs treatment after three weeks of chronic exposure.

Later, we analyzed the regulation of two isoforms of cyclooxygenase (COX-1 and COX-2) in the zebrafish brain after the C_70_ NPs exposure. Cyclooxygenase plays a role to produce prostanoids like prostaglandins and thromboxanes that are all responsible for the inflammatory response. Interestingly, COX-1 expression was significantly elevated in the 1.5 ppm C_70_ NP-treated group compared to the untreated control and 0.5 ppm C_70_ NPs treated. The COX-2 protein was expressed at a very low level in the brain tissue of C_70_ NPs exposed to adult zebrafish compared to untreated control.

### 2.8. Clustering of Zebrafish Behavior Distinguished Carbon NPs (C_60_ and C_70_) Based on Their Exposure Concentration

**Figure 7:**
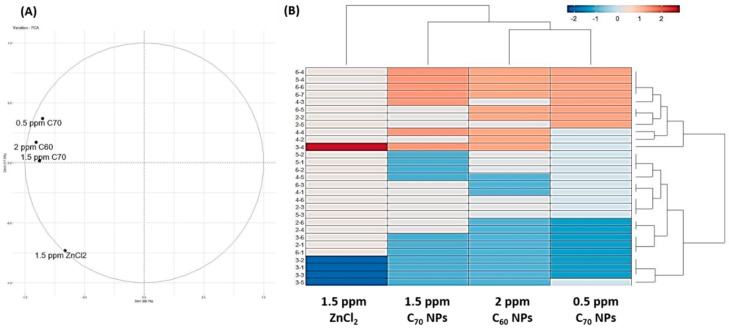
Comparison of behavioral alterations in zebrafish after exposing to either C_60_ NPs, C_70_ NPs or ZnCl_2_. (**A**) Results obtained from principal component analysis (PCA). (**B**) Results obtained from hierarchical clustering and heat map analysis.

Next, we would like to explore the difference of behavioral alteration between C_70_ NPs and other chemical toxicity by using a novel phenomic approach. In addition to the high and low doses of C_70_ fullerene NPs treatment, we also included our previous published C_60_ NPs [43] and ZnCl_2_ [15] data for cluster comparison. Initially, we transformed the behavioral endpoints for novel tank exploration, mirror biting, predator avoidance, social interaction, and shoaling into a scoring matrix. Later, this scoring matrix was subjected to principal component analysis (PCA) to elucidate the relationship between each experimental group. Both PCA (Figure 7A) and hierarchical clustering analysis (Figure 7B) demonstrate the behavioral alteration patterns between C_60_ and C_70_ NPs were close to each other and could be grouped into a single clade. The ZnCl_2_-exposed fish, on the contrary, displayed a distinct behavioral alteration pattern from those for the fullerene-exposed fish. For instance, ZnCl_2_ exposure can strongly increase the freezing behavior (behavioral endpoint 3-4) and reduce the average swimming speed (behavioral endpoint 3-1), mirror biting time percentage (behavioral endpoint 3-2), longest duration in the mirror side (behavioral endpoint 3-3), and swimming time movement ratio (behavioral endpoint 3-5) of the treated zebrafish when compared to C_60_/C_70_ NPs exposure. Based on behavioral phenomic evidence collected here, we concluded each chemical can induce unique, fingerprint-like behavioral alteration patterns in zebrafish.

## 3. Discussion

The biochemical and behavioral effects of fullerene C_70_ NPs were assessed in vivo using the adult zebrafish as a model organism. The result presented herein clearly demonstrates the usefulness of this model as an effective platform to rapidly assess manufactured fullerene nanomaterial toxicity. Thus far, much of the data on the effects of fullerene exposure had been obtained from in vitro methods [15,37,42,44,45] with few exceptions [37]. Research on C_70_ NPs toxicity is scarce. In vitro data may be of lacking the predictive ability of in vivo responses, particularly since those results might be dependent on the cell culture system selected for the experiment. For instance, the cytotoxicity of fullerene C_60_ to human liver carcinoma cells (HepG2), neuronal human astrocytes, and dermal fibroblasts were found to be dependent on cell types [46].

Recently, several reports indicated that nano-sized particulate matters can reach the brain and may be related to neurodegenerative diseases [47,48]. Another study in mice revealed that nanoparticles might be taken up to the brain from olfactory epithelium to the various parts of the rat brain through olfactory nerves [49]. Our results on adult zebrafish exposure to C_70_ NPs were similar to those observations and caused behavioral impairments, the deregulated levels of various biomarkers. These observations indicated that C_70_ NPs produced direct or indirect inflammations to the fish brain.

The present research is the first to demonstrate an environmentally relevant carbon-based nanoparticulate induces behavioral dysfunction in zebrafish. Here, we use the zebrafish model to show the effect of two different doses of C_70_ NPs on different behavior parameters including locomotion, exploration, shoaling, circadian rhythm, and social interaction, important stress-related biomarkers and neurotransmitters. Since animal behavior is considered as the result of complex interactions between a species and the environment, the pattern of the behavioral repertoire of a species could be used as an indicator of the health status of an organism [50]. Behavioral responses can have ecological effects at community and population levels [16]. Therefore, behavioral data could provide valuable information as early reporters of toxicity at higher levels of biological organization [51]. In this experiment, novel tank test results showed zebrafish chronically exposed to 1.5 ppm of C_70_ NPs presented significant alterations on total distance traveled, suggesting both motor and locomotor behavior patterns are impaired compared with the 0.5 ppm and untreated controls. In addition, in a circadian rhythm test, both of the C_70_ NP_-_treated groups also showed a significant decrease in locomotor activity in both light and dark cycles. Altered activity patterns and locomotion can lead to an increased vulnerability to predators [52]. Another changed parameter was aggressive behavior. Fish exposed to C_70_ NPs revealed a significant reduction in the aggressive nature of zebrafish, which plays a crucial role in the behavior and ecology of adult fish. Furthermore, C_70_ NPs were also found to dysregulate zebrafish social interaction behavior. Since this behavior is related to foraging, mating, fear response, and defense against predators, these behavioral deficits may also be related to the loose shoals formed observed during the shoaling test. These results showed that alterations in normal zebrafish behavior and impaired mobility in fish exposed to C_70_ NPs, which could compromise the survival of a population in natural environments. In addition, C_70_ NPs exposure was also found to change color preferences pattern. Together, behavioral response assessment may serve as a discerning tool for quantitative monitoring of toxicological effects of nanoparticle-based water contaminants in fish species [53].

Among the biomarkers of toxicity, our data showed a distinct production of ROS and lipid peroxidation (MDA content increased) in the muscle and gills of the fish treated with a higher dose of nanoparticles (Table 1), indicating that these C_70_ NP-treated fish experienced severe oxidative stress. Similarly, in another rodent study reported particulate matters in polluted air caused oxidative stress in the mouse brain [54]. The over-accumulation of ROS would tip the balance of the antioxidative/oxidative system in the brain, resulting in the significant reduction of the antioxidative enzymes such as SOD and CAT (Table 1). Nanoparticles were no longer freely circulated in the cytoplasm after being internalized by cells but were preferentially located in mitochondria [15]. However, when the mitochondria were invaded by the nanomaterials, the antioxidant defense capacity could be compromised [55]. Our study showed that the total anti-oxidation capacity decreased with increasing C_70_ NP doses. Muscle, gills, brains, and liver are organs with an active metabolism, responsible for vital functions of the body such as respiration, motion, behavior, excretion, and accumulation of xenobiotics [56]. Finally, it is noteworthy that due to its smaller size, and larger surface per mass and high reactivity, our data showed that C_70_ NPs were able to migrate into the brain more readily, were absorbed more from the circulation, and thus led to more severe toxicity in adult zebrafish than the bulk fullerene.

Recent trends in information technology have seen great momentum in biomedical research that helped usher in a new generation of approaches to understanding and sharing knowledge in both disciplines. Today, systems biology utilizes large-scale datasets to investigate molecular signaling networks from an integrative and comprehensive standpoint. Since the advent of proteomics, transcriptomics, and genomics, various data-rich fields of biology such as metabolomics and glycomics have emerged based on the compilation and validation of large-scale datasets. It is in this regard that the massive amount of phenotypic datasets generated by high-throughput behavioral screens has given rise to a new and vibrant field of a new subject neuro-phenomics: the integrative analysis of neural phenotypes and their regulation by various environmental and genetic factors [57]. With the wide application of zebrafish in neuroscience, a better understanding of the role of environmental factors in aquatic models facilitates further in-depth neuro-phenotyping studies by this small animal model [58]. The influence of various environmental pollutants/modifiers on zebrafish behavioral phenotypes is scarce [59]. To this end, we applied the phenomics’ approach; a cluster vector-based method was used following cross-calculated PCA and heatmap to highlight the main behavioral alterations induced by fullerene C_70_ NPs. In essence, this phenomics approach combines fast and simple data acquisition with complex and extensive behavioral analysis (enabled by behavior recognition, movement pattern, and video tracking), with ease in studies with a small sample size.

Most relevant to the present investigation is research in zebrafish embryos, which indicated a significant increase in pericardial edema, malformations, and mortality resulted after exposure to C_70_ NPs [60]. They directly determined in vivo fullerene exposures induced cellular death by two independent cellular death assays. We did not investigate any cell death assays but analyzed the behavioral changes and significant changes in biochemical assays that can provide important insights into the physiology of the organism. Up-regulation of proteins with antioxidant activity (including reactive oxygen species, MDA and TBARS) after fullerene C_70_ NPs treatments in the present study was consistent with the notion that the fish were responding to oxidative injury by activating a defense mechanism following exposure to nanoparticles. Oxidative stress is a crucial subject in aquatic toxicology. Damage to mitochondrial structure and function accelerates ROS production and causes oxidative stress [61]. The present study showed that C_70_ NPs could inhibit the activities of antioxidant enzymes, including CAT and SOD as confirmed by the biochemical assay in adult zebrafish tissues after exposure. These results demonstrated that C_70_ NPs elicited oxidative damages. In addition, the immune system plays an important role when assessing chemical toxicity. Previous reports have shown that chemicals can dysregulate the immune system and exert immunotoxicity on animals [34,62]. In the present study, the expression of TNF-α and IL-1β, representative proteins of inflammations, were obviously up-regulated at 1.5 ppm C_70_ NPs exposure. This phenomenon indicates that C_70_ NPs triggered a significant immune response suggesting a synergistic effect on inflammation. Inflammation and oxidative stress are concatenated processes that are usually activated in cells due to stress [63]. Consistent with the ROS measurement result, over-accumulation of inflammation is well known to contribute to the high level of ROS in the brain. ROS is produced through the Fenton reaction of amyloid Aβ with metal ions and causes the accumulation of inflammatory cytokines, including TNF-α and IL-1β that attract active plaques [64]. In addition, this result is in line with another prior study by Zhang and colleagues. In their study, it was found that the bare and starch-coated NPs displayed different tissue toxicity and both types of NP could induce inflammation and oxidative stress [65]. Moreover, the lack of anti-inflammatory function observed in the tissues was also related to the up-regulated ssDNA level. The ssDNA content in all treatment groups was higher than that of the untreated group. These results indicated that the DNA damage response was also activated after C_70_ NPS treatment. It might be interpreted as an adaptation during fullerene intoxication.

Adult zebrafish exposed to C_70_ NPs were not lethal, but behavioral changes and biochemical responses were obvious compared to untreated controls. Of all the biochemical markers tested, the majority of them showed alterations at the protein level and gills pathology. Further investigation is required to determine if these changes relate to corresponding gene expression or signal transduction pathways indicative of exposure to C_70_ NPs aggregates and changes in proteomic profiles. The behavioral impairments observed in zebrafish exposed to C_70_ NPs indicated that the exposure scenario used in this study had significant effects on the fish through ecologically-relevant doses used. Longer exposure to C_70_ NPs or other exposure scenarios (including different species, mode of exposure) may result in different effects. A fine but the significant point is that, in this study, changes in biochemical results were investigated after 21 days, presumably the expression of these proteins/enzymes could have been affected differently after different exposure durations and at different developmental stages of zebrafish.

## 4. Summary and Conclusions

**Figure 8:**
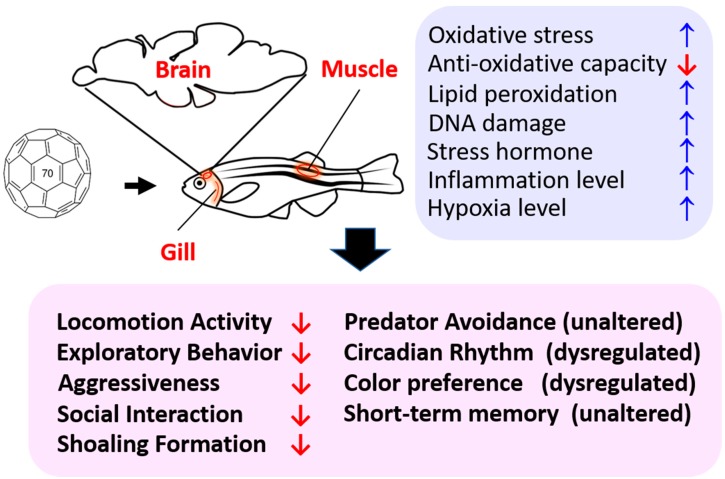
Schematic diagram showing the detrimental effects of chronic exposure of C_70_ nanoparticles on adult zebrafish. The corresponding behavioral alterations (pink color) and biochemical alterations in the brain tissue (blue color) after C_70_ nanoparticles exposure are summarized.

To our knowledge, this is the first study to show behavioral alterations induced by fullerene C_70_ NPs chronic exposure in adult zebrafish at environmentally-relevant concentrations. These effects seem to be correlated with changes in oxidative stress, inflammation, hypoxia, and imbalance of neurotransmitters in the brain (summarized in Figure 8). By utilizing this multiple behavioral test approach, our dynamic whole-animal model can be used to reveal the toxic potential of novel manufactured nanomaterials at the behavioral, physiological, and cellular levels. Furthermore, information obtained using this animal model system could be used as rapid feedback for engineers manufacturing novel nanomaterials, such that they can consider potential toxicity to favor the development of engineered nanoparticles with minimal toxicity.

## 5. Materials and Methods

### 5.1. Chemicals

C_70_ Fullerene (C_70_, 99.5% purity) was purchased from Sigma-Aldrich (St. Louis, MO, USA) (Cat no. 482994) and DMSO was purchased from Fisher scientific (Waltham, MA, USA). All the reagents used were analytical grade.

### 5.2. Animals

Adult wild-type zebrafish AB strain (*Danio rerio*) of both sexes (6–7 months old) were used in this study. Animals were acclimatized for at least one week before the experiments and were fed by lab-grown brine shrimps two times a day. The fish were maintained in a healthy condition and free of any signs of infections, and were used according to the guidelines for the care and use of laboratory animals by CYCU. All procedures in the present study were approved by the Animal Ethics Committee of the Chung Yuan Christian University (Approval ID 107030; approval date: 19/12/2018).

### 5.3. C_70_ NP_S_ Suspensions

Suspensions of C_70_ NPs in DMSO were prepared as in the previously described protocol with some modifications [66,67]. C_70_ NPs suspensions were further sonicated for one hour prior to use. To avoid the photoexcitation of C_70_ NPs, the whole exposure procedure was in the absence of light. However, overnight sonication was necessary to uniformly distribute in DMSO.

### 5.4. Characterization of C_70_ NPs

Prior to the exposure, C_70_ NPs were characterized for size, surface area, and structural properties by scanning electron microscope and X-ray diffraction methods. The stock solution dispersion was confirmed by SEM. However, without the use of SDS the C_70_ NPs were not dispersed and clearly visible as polymerized long strands about 50–70 nm thick (Figure 1B). The C_70_ NPs were suspended in 0.1% DMSO, stirred well and then sonicated overnight, pipetted in 20ul droplets, deposited on a copper grid, and the sample grid was dried in a microwave oven for about 4 h without vacuum. Then the copper grid was directly inserted into a FESEM machine after it was completely dried. The images were taken with a 10k magnification CCD camera. The fullerene C_70_ NPs were further analyzed by the X-ray diffraction method and determined to be of high purity. C_70_ NPs dispersed in DMSO and stock solutions were made at concentrations of 0.5 mg/L and 1.5 mg/L. Samples of these suspensions were taken for analysis of particle size distribution, zeta potential, and particle dissolution. Particle size distributions were obtained using a Zetasizer Nano ZS with a 633 nm red laser and were capable of both particle size analysis (using dynamic light scattering as the basic principle of operation) and zeta potential measurement (Zeta Potential Instruments Inc., Long Island, NY, USA).

### 5.5. Zebrafish Exposure Protocol

The experimental adult zebrafish of males and females with an average age group of 6 months, an average weight of 0.60 ± 0.10 g, and an average length of 43.25 ± 2.76 mm, were selected for the study. Two different concentrations of C_70_ NPs suspensions (0.5 and 1.5 mg/L) were prepared prior to use. The fish were separated into three groups, each consisting of 20 adult zebra fish. The three groups were exposed to tank water (as a control), 0.5 ppm C_70_ NPs, and 1.5 ppm C_70_ NPs for a test period in 20 L glass tanks containing 15 L of a test solution. In order to maintain the constant concentration of the C_70_ NPs, the test suspensions were replaced every 24 h. For the behavior toxicity test, the control fish were not exposed to any nanoparticle or solvent, while the experimental group was treated with C_70_ NPs at different doses and about 70 % water was changed every 24h with refilling after each change. All fish were terminated at the end of the particle exposure and were sacrificed within minutes by immersed in the working concentration of tricaine (160 ppm) prior to body weight measurement. Once all the behavioral tests were done, the fish were anesthetized and immediately euthanized by immersion in high-dose tricaine solution at 1600 ppm (Sigma) and their tissues were removed for further biochemical assays. From each fish, brain, muscle, and gill tissues were independently harvested and all biochemical assays were performed. Exposure experiments were conducted three times.

### 5.6. Behavioral Endpoints

The behavioral endpoints being measured were the following: 3D locomotion, novel tank exploration, aggression, predator avoidance test, shoaling, conspecific social interaction, circadian rhythm, color preferences, and short-term memory tests. For 3D locomotion, novel tank exploration, aggression, predator avoidance test, shoaling, conspecific social interaction, and shoaling behaviors, the camera was placed around 5 m in front of the zebrafish tower which is described in our previously published protocol [68,69]. The video was recorded in black and white mode with a frame rate of 50 fps (frames per second).

#### 5.6.1. 3D Locomotion Test

3D locomotion test was performed on the 14th day of C_70_ NPs exposure to the zebrafish. The tracking procedure of C_70_ NP-treated fish was followed by our previously published method [70]. Two different concentrations of fullerene C_70_ NPs were used for this study (0.5 and 1.5 mg/L). Three separate experiments were performed using the same batch of C_70_ NP-exposed fish.

#### 5.6.2. Novel Tank Test

The novel tank test was defined by our previous publication [70] which reflected the congenital characteristics in the swimming behavior of zebrafish. Normally, zebrafish have two behavioral phenomena: freezing, which was defined as a total absence of movement, except for the gills and eyes for 1s or longer; and erratic movements, which was defined as sharp changes in direction or velocity and repeated darting behaviors. In this experiment, zebrafish were placed individually in the tank. Their behaviors were recorded for 1 min at intervals of 0, 5, 10, 15, 20, 25, and 30 min. A video camera with a long-range zoom lens feature was positioned in front of the test tank. The novel tank test parameters of behaviors were the following: average speed (cm/s), freezing time movement ratio (%), time in top duration (%), number of entries to the top, latency to enter the top (s), and total distance traveled in the top (cm). Later, the recorded videos were analyzed by idTracker [70] and data tracking was calculated using Microsoft Excel.

#### 5.6.3. Aggression Test

The aggression test was referenced from the previous study [70,71,72] with some modifications. C_70_ NP-treated adult fish and untreated fish were placed into the test tank containing a mirror placed on vertically to one side of the wall. The aggressive test parameters were the following: average speed (cm/s), mirror biting time percentage (%), longest duration in the mirror side (%), freezing, swimming, and rapid time movement ratio (%).

#### 5.6.4. Predator-Avoidance Test

The predator-avoidance behavior test of zebrafish was referenced from our previous publication [70]. The fear and escape behaviors as a response for the predator presence were determined by measuring average speed (cm/s), predator approaching time (%), the average distance to a separator (cm), freezing, swimming, and rapid movement ratio (%).

#### 5.6.5. Social Interaction Test

The social interaction test which was based on our previous publication was conducted to assess zebrafish social interaction behavior with their conspecifics [70]. In this assay, we measured interaction time percentage (%), longest duration in separator side (%), average speed (cm/s), and average distance to a separator (cm).

#### 5.6.7. Shoaling Test

The sixth zebrafish behavioral test in this experiment was the shoaling test, an assessment of group affiliation behavior. This test was conducted based on our previous publication [70]. In this test, average speed (cm/s), time in top duration (%), average shoal area (cm^2^), average inter-fish distance (cm), and average nearest and farthest neighbor distance (cm) were calculated.

#### 5.6.8. Circadian Rhythm Test

The circadian rhythm test was carried out to evaluate zebrafish sleep/wake behaviors on the 21st day of C_70_ NPs exposure and the test was based on previous publications [70]. In this experiment, we recorded zebrafish locomotor activity (average speed (cm/s), average angular velocity (°/s), and meandering (°/m)) for 60 s every 60 min in 24 h. Later, idTracker software was used to track fish movement trajectories.

#### 5.6.9. Color Preferences Test

The zebrafish color preferences were assessed using a 20 × 20 × 10 cm tank divided into a two-color partition (blue–red, blue–yellow, blue–green, red–yellow, red–green, and yellow–green). The experiment was recorded using a CCD camera (ONTOP, M2 module, China) for 30 min. The video was analyzed using idTracker to determine the position of the zebrafish.

#### 5.6.10. Short-Term Memory Test

We performed a short-term memory test by using a passive avoidance setting according to our previous publication [73,74]. Initially, 20 fish were randomly grouped into control and C_70_ NPs exposed groups with 10 fish each. Later, experimental group fish exposed to 1.5 ppm C_70_ NPs were exposed to a shuttle box to perform short-term memory tests. The learning latency, total number of electric shocks given for training, and memory latency were recorded for comparison between control and 1.5 C_70_ NP-exposed fish.

### 5.7. Total Protein Extraction

After behavioral tests, nine fish per treatment were collected from each tank for biochemical analysis. Gills, brain, and red muscle tissues were carefully removed and a pool of three fish was used for homogenate preparation. Tissues were homogenized at 8000 rpm in a bullet blender with 50 volumes of (*v*/*w*) ice-cold phosphate saline buffer with a pH of 7.2. Samples were further centrifuged at 4000 rpm for 20 min and the crude homogenate was stored in 100 µL aliquots at −20 °C until required. Total protein concentration in tissues was determined using a Pierce BCA Protein Assay kit (23225, Thermo Fisher Scientific, Massachusetts, MA, USA). The color formation was analyzed at 562 nm using a microplate reader (Multiskan GO, Thermo Fisher Scientific). Exposed fish tissues were analyzed to determine the possible effects of lipid peroxidation, oxidative stress, neurotransmitter changes, and antioxidant activity.

### 5.8. Biochemical Parameter Assay

To evaluate the toxic effects of C_70_ NPs, alterations of biomarkers in zebrafish tissues due to NPs exposure were determined. The tissue oxidative stress marker like reactive oxygen species was measured by ELISA kits purchased from a commercial company (ZGB-E1561, Zgenebio Inc., Taipei, Taiwan). The DNA damage marker (ssDNA) and lipid peroxidation (malondialdehyde, MDA, and thiobarbituric acid reactive substances—TBARS) markers were measured by target-specific ELISA kits purchased from a commercial company (ZGB-E1595, ZGB-E1592, and ZGB-E1605 Zgenebio Inc., Taipei, Taiwan). Cortisol, a stress hormone, and two inflammation markers of TNF-α and IL-1β were measured by using commercial target-specific ELISA kits (ZGB-E1560, ZGB-E1612, and ZGB-E1609, Zgenebio Inc., Taipei, Taiwan). Creatine kinase (CK) and adenosine-5′–triphosphate (ATP), keys marker for energy metabolism, and hypoxia-inducible factor 1-alpha (Hif1-α), a key marker for hypoxia, were measured by using target-specific ELISA kits purchased from a commercial company (ZGB-E1646, ZGB-E1645, and ZGB-E1643, Zgenebio Inc., Taipei, Taiwan). Cyclooxygenase (COX-1 and COX-2), enzymes for prostanoids production, were measured by target-specific ELISA kits purchased from a commercial company (ZGB-E1655 and ZGB-E1656, Zgenebio Inc., Taipei, Taiwan). The antioxidant enzymes-related biomarkers, like superoxide dismutase (SOD, ZGB-E1604) and catalase (CAT, ZGB-E1598), and neurotoxic responses in terms of acetylcholine esterase (AChE, ZGB-E1637), dopamine (DA, ZGB-E1573), acetylcholine (ACh, ZGB-E1585), melatonin (ZGB-E1597), and serotonin (ZGB-E1572) activities were measured by target-specific ELISA kits purchased from commercial company (Zgenebio Inc., Taipei, Taiwan) according to the manufacture’s protocol. These assay kits are based on the sandwich ELISA method that involves a specific antibody for the detection of the molecules of interest. Briefly, 10 µL of brain homogenate was placed onto the well. Then, 100 ul of horseradish peroxidase (HRP)-conjugate reagent was added into each well, covered with an adhesive strip and incubated for 60 min at 37 °C. The well was washed with wash solution (400 µL) and chromogen A 50 ul and chromogen B 50 µL were added to each well. The mixture was incubated for 15 min at 37 °C. Then the reaction was stopped by adding 50 µL of stop solution to each well. The absorbance was analyzed at 450 nm using a microplate reader (Multiskan GO, Thermo Fisher Scientific) within 15 min. The data were expressed as either U/µg, ng/µg, or pg/µg of total protein.

### 5.9. Statistical Analysis

The zebrafish behavioral data were analyzed by different types of statistical analysis. In the novel tank test, two-way ANOVA with Geisser–Greenhouse correction was conducted and the significant differences between the control group and C_70_ NP-treated groups during the whole 30 min test were described by the “*” symbol. For the 3D locomotion, mirror biting, predator avoidance, social interaction, shoaling, and circadian rhythm tests, the Kruskal–Wallis test with Dunn’s multiple comparisons test as a follow-up test was used. The color preference data were analyzed using two-way ANOVA followed by a Tukey post-hoc test. If the data were not normally distributed, they were analyzed using non-parametric Kruskal–Wallis followed by Dunn’s post-hoc test. The biochemical data were analyzed individually (*n* = 20 for both the control fish group and C_70_ nanoparticle-treated fish group. Biomarker responses of exposed fish were compared with control fish by a one-way ANOVA test followed by the post-hoc test of Tukey, depending upon the data normality for significant data. The level of significance was set at a *p* value <0.05. All statistics were plotted and compiled by using GraphPad Prism (GraphPad Software version 7 Inc., La Jolla, CA, USA).

### 5.10. PCA, Heatmap, and Clustering Analysis

All behavior results data were converted in to excel file using Microsoft Excel. Every endpoint alteration was defined as a number, ranging from −4 to 4, where −4 means the value of the endpoint was significantly lower than the control group (**** *p* < 0.0001) and 4 means the endpoints values was significantly higher than the control group (**** *p* < 0.0001). If the treated fish behavioral endpoint was not significantly different from the control fish, the number was defined as 0 (*p* > 0.05). All of the important behavioral endpoints in each test were listed in the previous study [42]. Next, the excel file was converted to a comma delimited type file (.csv) in order to be readable by R software. PCA, heatmap, and clustering analysis were carried out by R software (https://www.r-project.org/).

## Figures and Tables

**Table 1:** Comparison of biomarker expression in the muscle, brain, and gill tissues in C_70_ NP-exposed zebrafish. Data are expressed as the mean ± SEM.

Biomarker	WT (*n* = 10)	C70 (0.5 ppm)	C70 (1.5 ppm)	Unit	Significance	ANOVA F Value	*p* Value
**Muscle**							
ROS	2.95 ± 0.12	5.62 ± 0.26 ***	8.46 ± 0.25 ****	U/ug of total protein	YES	F (2, 6) = 157.2	*p* < 0.0001
CAT	3.03 ± 0.13	5.01 ± 0.44 **	3.82 ± 0.01 ^NS^	U/ug of total protein	YES	F (2, 6) = 14.10	*p* = 0.0054
SOD	6.19 ± 0.78	14.38 ± 0.34 ***	14.34 ± 0.54 ***	U/ug of total protein	YES	F (2, 6) = 66.33	*p* < 0.0001
TBARS	5.60 ± 0.34	7.39 ± 0.36 *	8.37 ± 0.29 **	ng/ug of total protein	YES	F (2, 6) = 17.92	*p* = 0.0029
MDA	0.15 ± 0.00	0.20 ± 0.01 *	0.22 ± 0.01 *	ng/ug of total protein	YES	F (2, 6) = 8.826	*p* = 0.0163
Cortisol	17.95 ± 0.90	20.87 ± 1.17 ^NS^	23.95 ± 0.66 **	pg/ug of total protein	YES	F (2, 6) = 10.3	*p* = 0.0114
Hif1-α	11.65 ± 0.54	17.60 ± 0.87 **	18.45 ± 1.00 **	pg/ug of total protein	YES	F (2, 6) = 20.05	*p* = 0.0022
ssDNA	0.61 ± 0.01	0.81 ± 0.07 *	0.89 ± 0.05 **	U/ug of total protein	YES	F (2, 6) = 9.408	*p* = 0.0141
TNF-α	6.86 ± 0.41	8.90 ± 0.33 **	11.76 ± 0.26 ***	pg/ug of total protein	YES	F (2, 6) = 52.24	*p* = 0.0002
IL1-β	0.42 ± 0.05	0.47 ± 0.02 ^NS^	0.57 ± 0.03 *	ng/ug of total protein	YES	F (2, 6) = 5.946	*p* = 0.0377
ATP	363.60 ± 9.02	343.60 ± 15.23 ^NS^	282.20 ± 9.30 **	ng/ug of total protein	YES	F (2, 6) = 13.5	*p* = 0.0060
CK	3.06 ± 0.14	4.78 ± 0.34 **	2.79 ± 0.14 ^NS^	pg/ug of total protein	YES	F (2, 6) = 22.17	*p* = 0.0017
**Brain**							
ROS	0.90 ± 0.03	1.61 ± 0.19 ^NS^	3.53 ± 0.64 **	U/ug of total protein	YES	F (2, 6) = 12.38	*p* = 0.0074
SOD	2.20 ± 0.06	3.44 ± 0.08 ****	3.28 ± 0.09 ***	U/ug of total protein	YES	F (2, 6) = 73.1	*p* < 0.0001
Cortisol	3.47 ± 0.13	4.76 ± 0.08 *	4.91 ± 0.51 *	pg/ug of total protein	YES	F (2, 6) = 6.561	*p* = 0.0309
Hif1-α	4.26 ± 0.11	4.91 ± 0.16 ^NS^	6.86 ± 0.37 ***	pg/ug of total protein	YES	F (2, 6) = 32.09	*p* = 0.0006
ssDNA	0.13 ± 0.01	0.28 ± 0.09 ^NS^	0.37 ± 0.02 *	U/ug of total protein	YES	F (2, 6) = 5.274	*p* = 0.0477
ACh	4.44 ± 0.15	4.61 ± 0.08 ^NS^	2.93 ± 0.31 **	U/ug of total protein	YES	F (2, 6) = 20.92	*p* = 0.0020
AChE	0.70 ± 0.10	0.77 ± 0.02 ^NS^	1.03 ± 0.08 **	U/ug of total protein	YES	F (2, 6) = 12.75	*p* = 0.0069
Melatonin	1.02 ± 0.03	0.94 ± 0.04 ^NS^	0.68 ± 0.03 ***	pg/ug of total protein	YES	F (2, 6) = 31.66	*p* = 0.0006
Serotonin	0.20 ± 0.01	0.22 ± 0.01 ^NS^	0.14 ± 0.00 **	ng/ug of total protein	YES	F (2, 6) = 31.05	*p* = 0.0007
Dopamine	9.04 ± 0.08	9.30 ± 0.20 ^NS^	6.92 ± 0.09 ****	pg/ug of total protein	YES	F (2, 6) = 95.27	*p* < 0.0001
COX-1	0.16 ± 0.00	0.18 ± 0.00 ^NS^	0.38 ± 0.01 ****	U/pg of total protein	YES	F (2, 6) = 258.2	*p* < 0.0001
COX-2	0.74 ± 0.01	0.94 ± 0.12 ^NS^	0.64 ± 0.03 ^NS^	U/pg of total protein	YES	F (2, 6) = 4.32	*p* = 0.0688
**Gills**							
CAT	1.44 ± 0.03	1.64 ± 0.02 **	1.30 ± 0.03 *	U/ug of total protein	YES	F (2, 6) = 44.21	*p* = 0.0003
TBARS	1.71 ± 0.14	1.64 ± 0.06 ^NS^	2.30 ± 0.68 ^NS^	ng/ug of total protein	NO	F (2, 6) = 0.821	*p* = 0.4842
MDA	0.08 ± 0.00	0.09 ± 0.00 ^NS^	0.11 ± 0.00 **	ng/ug of total protein	YES	F (2, 6) = 21.29	*p* = 0.0019
TNF-α	3.06 ± 0.10	2.98 ± 0.03 ^NS^	8.68 ± 0.74 ***	pg/ug of total protein	YES	F (2, 6) = 56.94	*p* = 0.0001

N.S. = not significant, * *p* < 0.05, ** *p* < 0.01, *** *p* < 0.001, **** *p* < 0.0001.
